# Supplementary material for: Natural variation of macrophage activation as disease-relevant phenotype predictive of inflammation and cancer survival
Source: Nat Commun. 2017 Jul 24;8:16041. doi: 10.1038/ncomms16041 (PMC5527282; doi:10.1038/ncomms16041)
Supplement: Supplementary Information [file ncomms16041-s1.pdf]

Type of file: PDF
Title of file for HTML: Supplementary Information
Description: Supplementary Figures, Supplementary Table and Supplementary References

Type of file: XLSX
Title of file for HTML: Supplementary Data 1
Description: List of classical and recombinant mouse strains of the HMDP ranked according to the polarization factor ratio (PFR) based on iNOS or IL-12beta as defined in figure 2.

Type of file: XLSX
Title of file for HTML: Supplementary Data 2
Description: Full gene list of M(LPS)+/- signatures as defined in figure 3.

Type of file: XLSX
Title of file for HTML: Supplementary Data 3
Description: Predicted upstream transcription factors of M(LPS)+/- signatures using Ingenuity.

Type of file: PDF
Title of file for HTML: Peer Review File

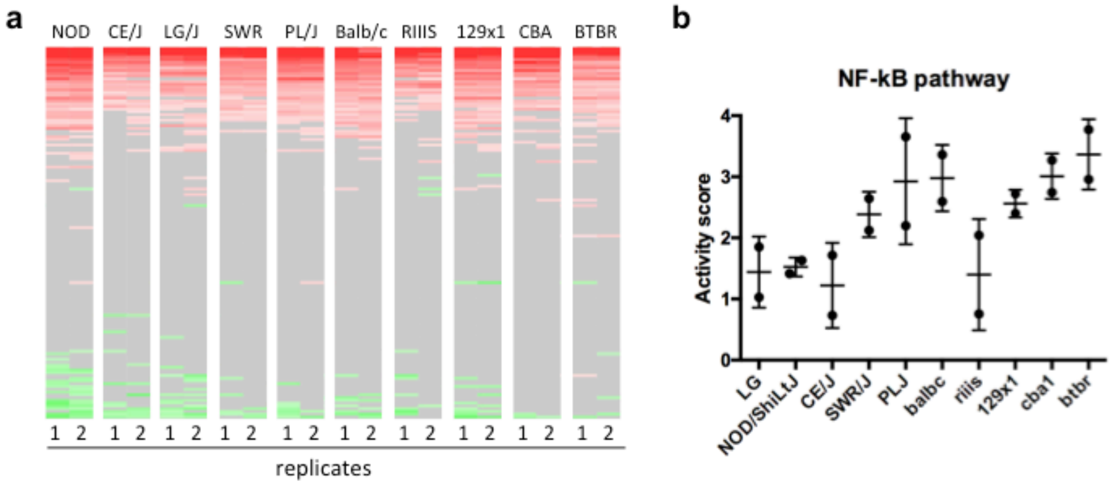

Supplementary Figure 1. NF-κB pathway activation of biological replicates. Related to Figure 1. a,b) The NF-κB pathway activation was determined in macrophage transcriptomes by IPA using biological replicates of classical mouse strains. Mean and S.D. indicated.

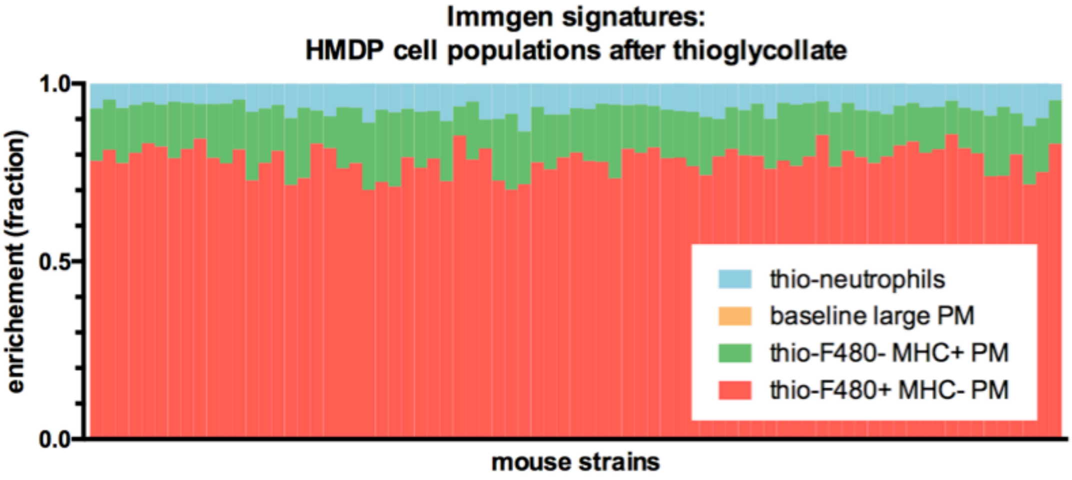

Supplementary Figure 2. The cellular composition in the peritoneal cavity is mostly similar in all mouse strains after 5 days thioglycollate treatment. RNA deconvolution (Cibersort) of the full transcriptome of the hybrid mouse diversity panel (83 strains) using Immgen reference signatures (GSE15907) of different cell types in the peritoneal cavity (PC). Blue = neutrophils in the thioglycollate-treated PC. Orange = large peritoneal macrophages in the untreated PC were not detected. Green and red = two macrophage populations in the thioglycollate treated PC. Thioglycollate treatment for 5 days. PM = peritoneal macrophages.

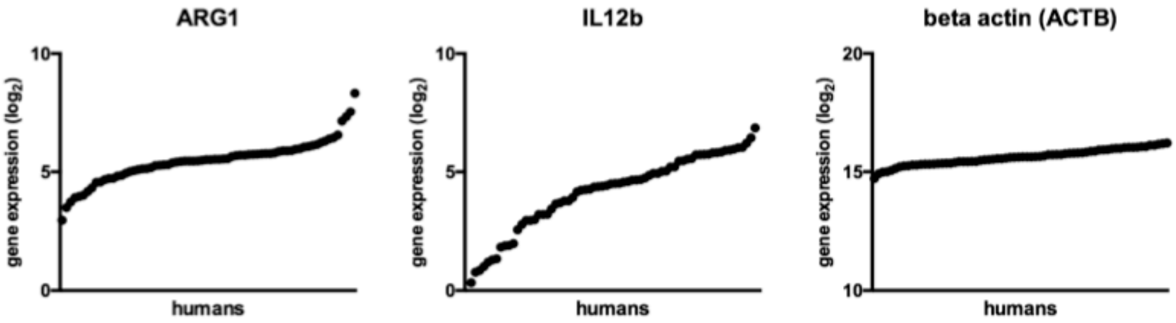

Supplementary Figure 3. Macrophage gene diversity in 70 humans. Related to Figure 1. Variation of gene expression (Arginase and Interleukin 12 beta) in human alveolar macrophages from 70 different donors (bronchoalveolar lavage). The ACTB gene is shown as control. Data from GSE13896.

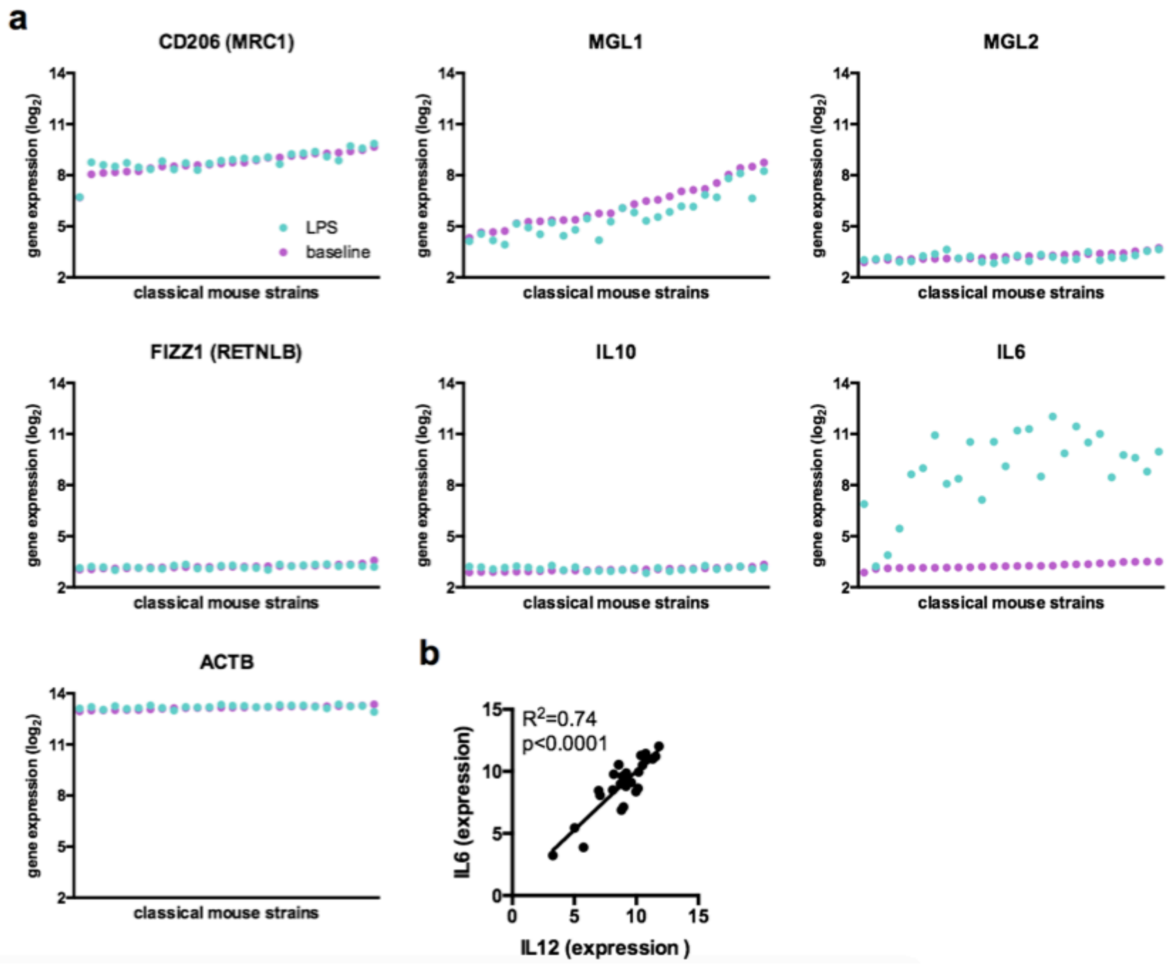

Supplementary Figure 4. Expression of key macrophage genes in the HMDP. Related to Figure 1. a) Gene expression at baseline (purple) and after LPS treatment (cyan) for select genes across the classical inbred strains of the mouse diversity panel. All strains are ranked by the baseline value for each gene. b) IL-6 and IL-12b gene expression (as $\log_2$ RMA) of LPS treated macrophage transcriptome highly correlate. $R^2 = 0.74$, $p < 0.0001$.

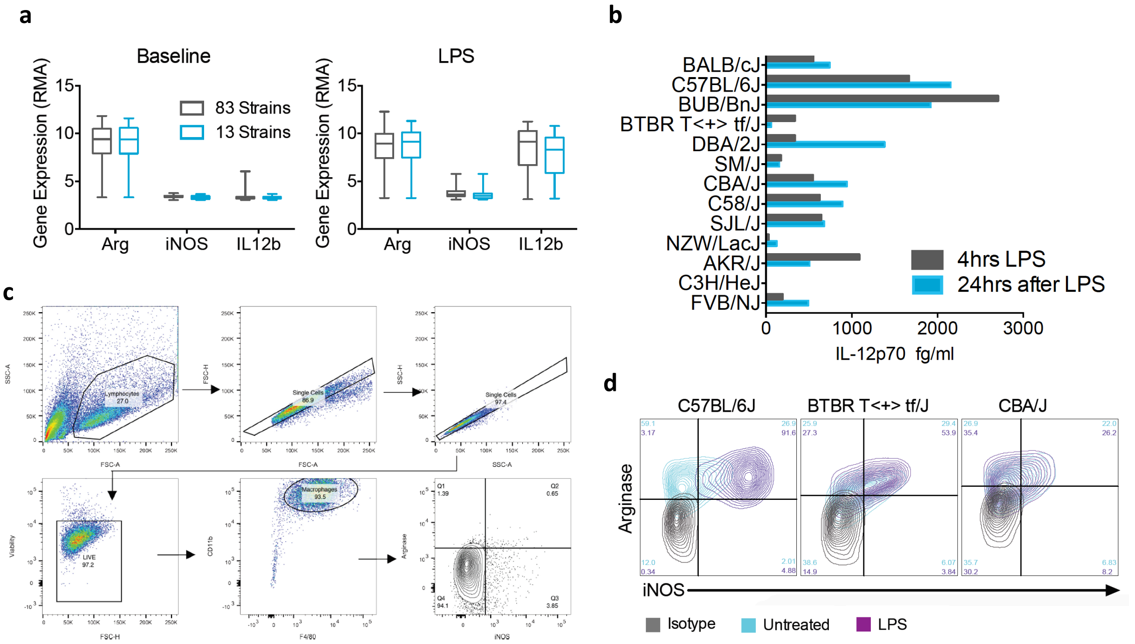

Supplementary Figure 5. Protein expression varies in 13 representative strains of the HMDP. a) Box plots of average, top and bottom quartile and extremes of Arginine, iNOS (NOS2), and IL-12β gene expression (RMA) in peritoneal macrophages of all 83 analyzed strains and a representative selection of 13 strains (Balb/c, C57BL/6J, BuB/BnJ, BTBR T<+> tf/J, DBA/2J, SM/J, CBA/J, C58/J, SJL/J, NZW/LacJ, AKR/J, C3H/HeJ, FVB/NJ) at baseline and after LPS challenge. b) IL-12 p70 (p35/p40 heterodimer) measured by cytometric bead array in supernatants of peritoneal macrophages after 4 hour LPS stimulation (black bars) and 24 hours after LPS (blue bars). IL-12 p70 was undetectable in all strains without LPS and remained undetectable in C3H/HeJ. c,d) Peritoneal macrophages were harvested from the 13 selected strains and analyzed using flow cytometry as outlined. They were left untreated (baseline, blue) or treated with LPS for 4 hours in vitro (LPS, purple) and stained for iNOS (x axis) and arginase-1 (y axis). Isotype controls in black. All strains showed one of the 3 representative patterns shown.

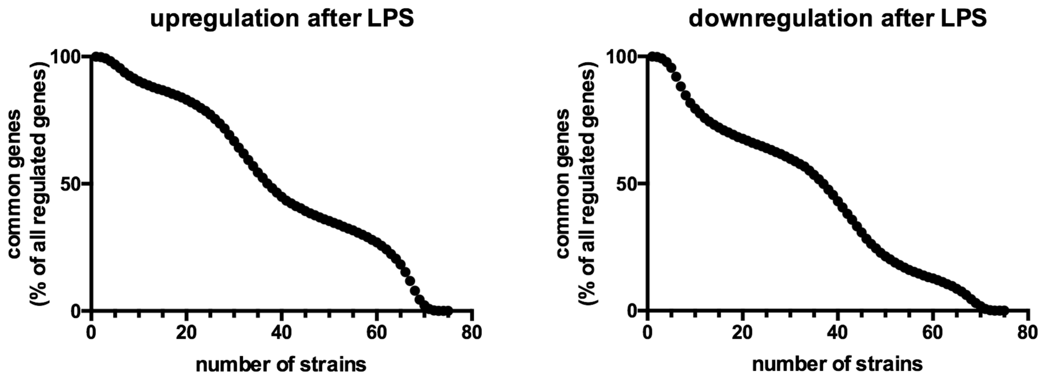

Supplementary Figure 6. No common LPS gene signature can be detected across all mouse strains. The up- and down regulated genes in response to LPS for each mouse strain were calculated (log-fold change of > 2), and for each gene the overlap with all other strains was determined. 100% equals all LPS regulated genes in all strains. As example, only 26.9% of the total upregulated genes are shared by 60 strains, and 83% of the upregulated genes are shared by 20 strains. The more strains are analyzed the less the overlap of commonly regulated genes. No common genes can be detected in 74 or more mouse strains.

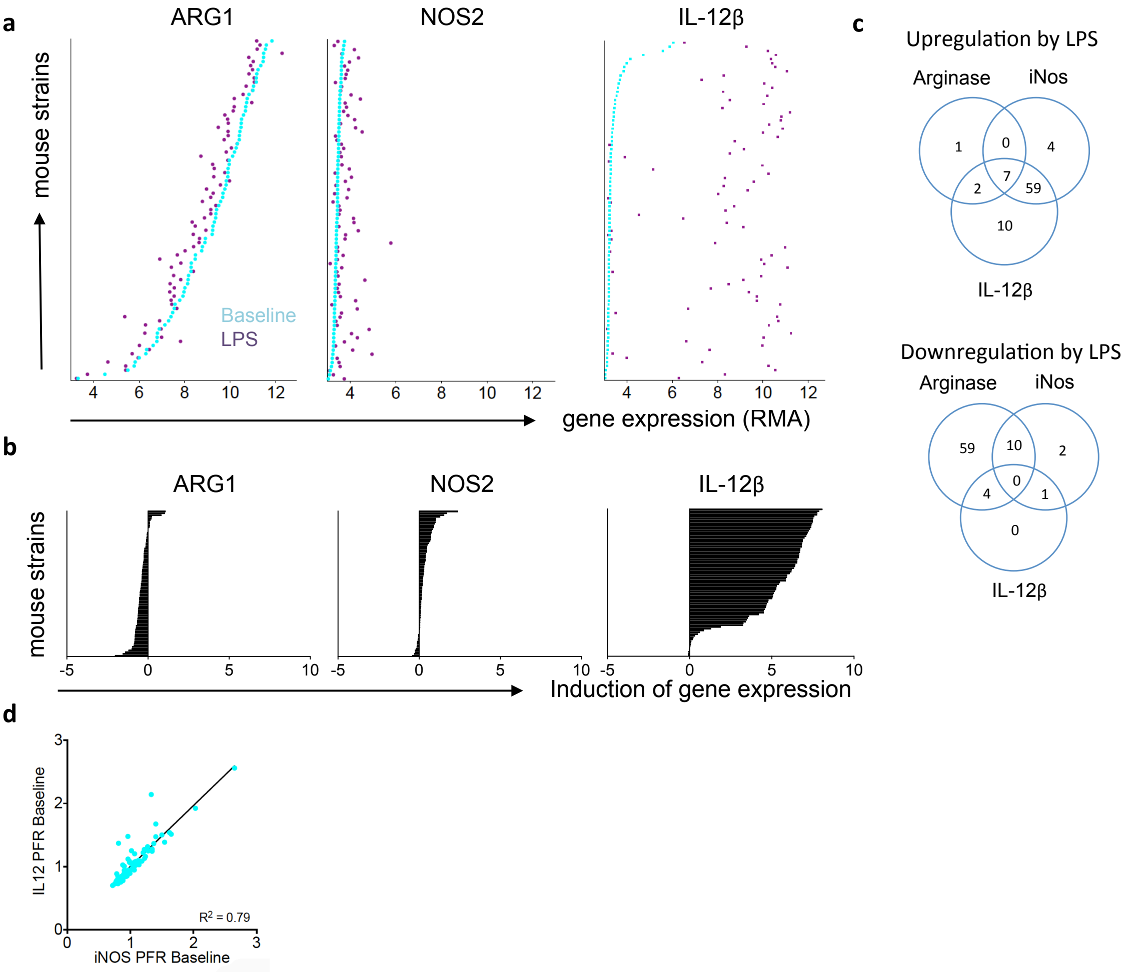

Supplementary Figure 7. Comparison of NOS2 and IL12β upregulation across the HMDP. Related to Figure 2. a) Arginine, iNOS (NOS2), and IL-12β gene expression as RMA (quantile normalized $\log_2$) in peritoneal macrophages for all analyzed 83 strains of the hybrid mouse diversity panel. Each mouse strain is represented by a dot at baseline (cyan) and after LPS challenge *in vitro* (purple). b) Induction plots of Arginine, iNOS (NOS2) and IL-12β in peritoneal macrophages of 83 mouse strains after LPS challenge *in vitro* compared to baseline gene expression. Each plot is ordered ascendingly. c) Venn diagrams categorizing 83 mouse strains according to their up- (top panel) or downregulation (bottom panel) after LPS challenge for Arginine, iNOS (Nos-2) and IL-12β. d) Correlation of the polarization factor based on iNOS and IL-12β gene expression as outlined in Figure 2.

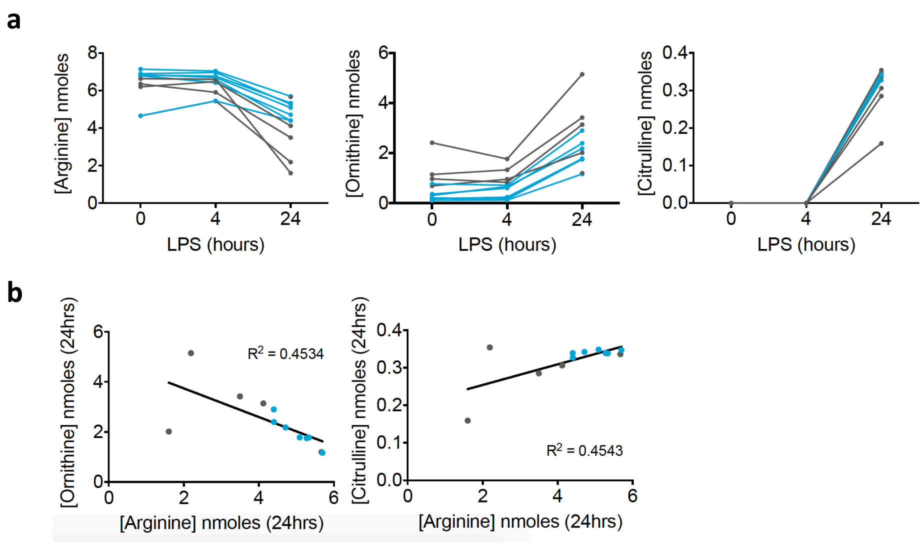

Supplementary Figure 8. Profiling of macrophage metabolites after LPS treatment. a) Enzymatic product levels of arginine, ornithine and citrulline in macrophage supernatant collected at baseline, 4 or 24 hours after LPS, measured by amino acid analysis for 13 selected strains (see suppl. Figure 5). Citrulline was not detectable at baseline and 4h. LPS-responsive and -unresponsive mouse strains as determined by the IL12β/Arg-1 polarization factor are shown in blue and gray, respectively. b) Correlations of citrulline and ornithine with arginine ($p < 0.0162$ and 0.0164, respectively) at 24h after LPS. No correlation between citrulline and ornithine (data not shown).

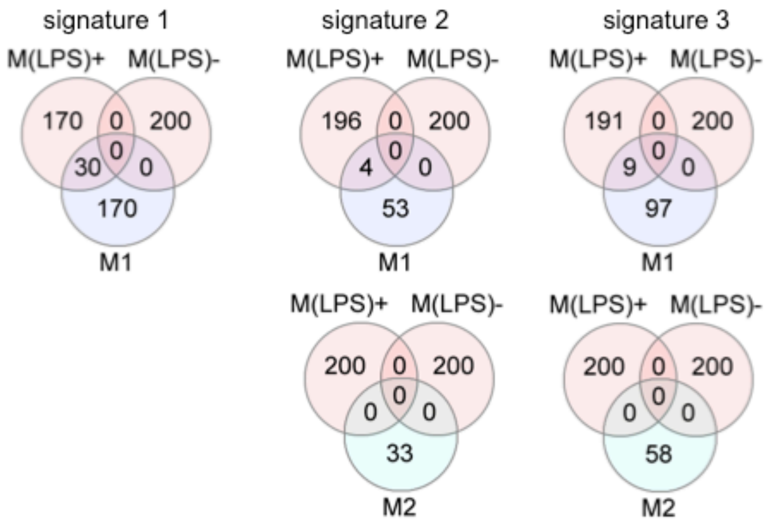

Supplementary Figure 9. Comparison of M(LPS)+/- signatures to published M1 macrophage gene signatures. Overlap between 3 different published macrophage activation gene signatures and our LPS-responder/non-responder (M(LPS)+/-) gene lists. Details about the signatures 1-3 can be found in Supplementary Data 3. In signature 1 there is no M2 gene set available.

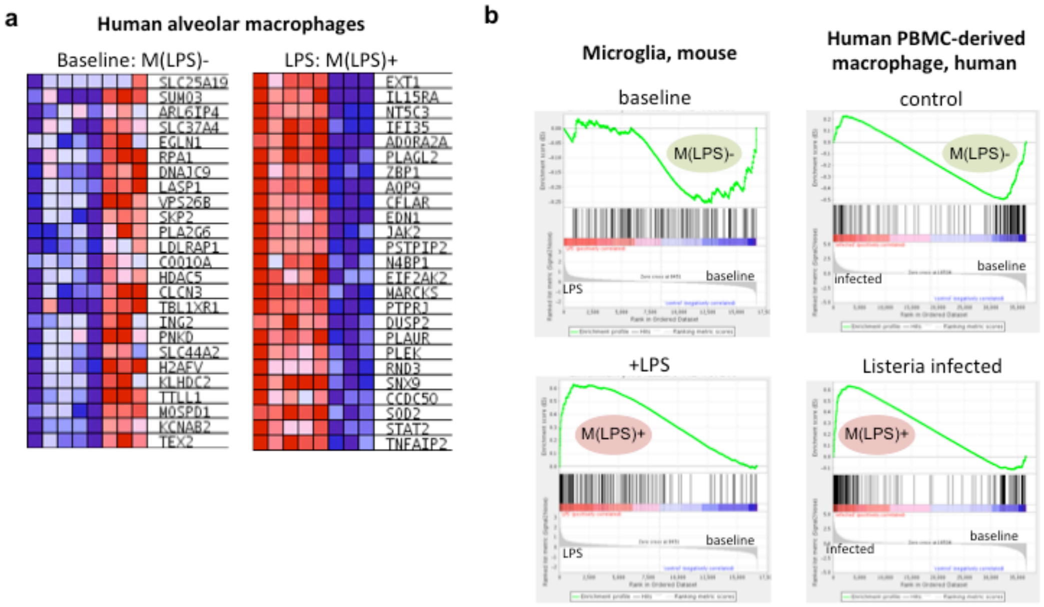

Supplementary Figure 10. Gene set enrichment analysis of M(LPS)$^{+/-}$ signatures in mouse and human macrophage data sets. Related to Figure 4. a) Heatmaps of enriched M(LPS)$^{+}$ or M(LPS)$^{-}$ genes detected in human alveolar macrophages treated with saline or LPS intratracheally. The enrichment of M(LPS)$^{+}$ and M(LPS)$^{-}$ macrophage gene signatures in saline or LPS treated human alveolar macrophages was determined using GSEA (gene set enrichment analysis). Leading edge genes are shown as heatmap. Red = upregulation, blue = downregulation. Data from GSE40885. Every column represents one sample (baseline: n = 3, LPS: n = 5) b) GSEA of macrophage gene signatures in isolated mouse microglia at baseline and after intracerebral LPS-injection. Data from GSE67858. c) GSEA of macrophage gene signatures in CD14^{+}CD68^{+} macrophages derived from human peripheral blood mononuclear cells (PBMC) with or without Listeria monocytogenes infection. Data from GSE34103.

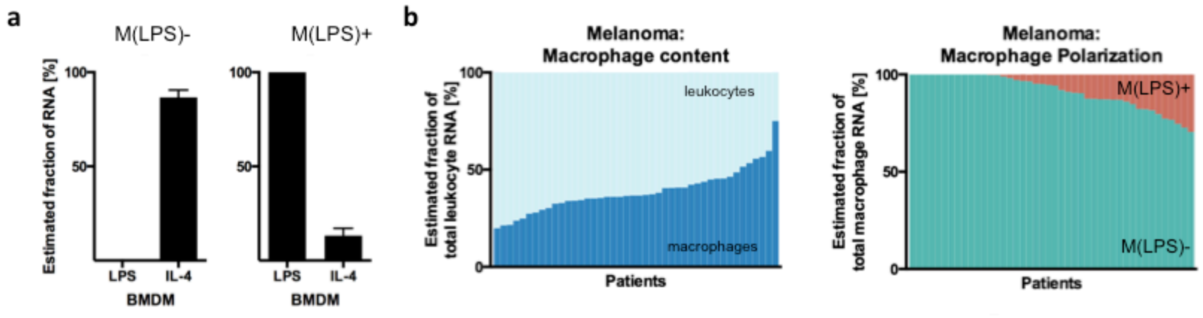

Supplementary Figure 11. RNA deconvolution using M(LPS)$^{+/-}$ signatures. Related to Figure 5. a) Validation of the CIBERSORT [1] deep deconvolution algorithm using the transcriptome of mouse bone marrow derived macrophages (BMDM) treated with LPS (M1 polarization) or IL-4 (M2 polarization). Top 400 genes for M1 and M2 were used as input signature. Data from GSE68167. Mean and S.D. indicated b) Estimation of the monocyte/macrophage content and the macrophage polarization in bulk melanoma biopsies using CIBERSORT deconvolution and the provided LM22 leukocyte or the developed M1/2 signatures. Melanoma data (n = 44 patients) is from GSE19234.

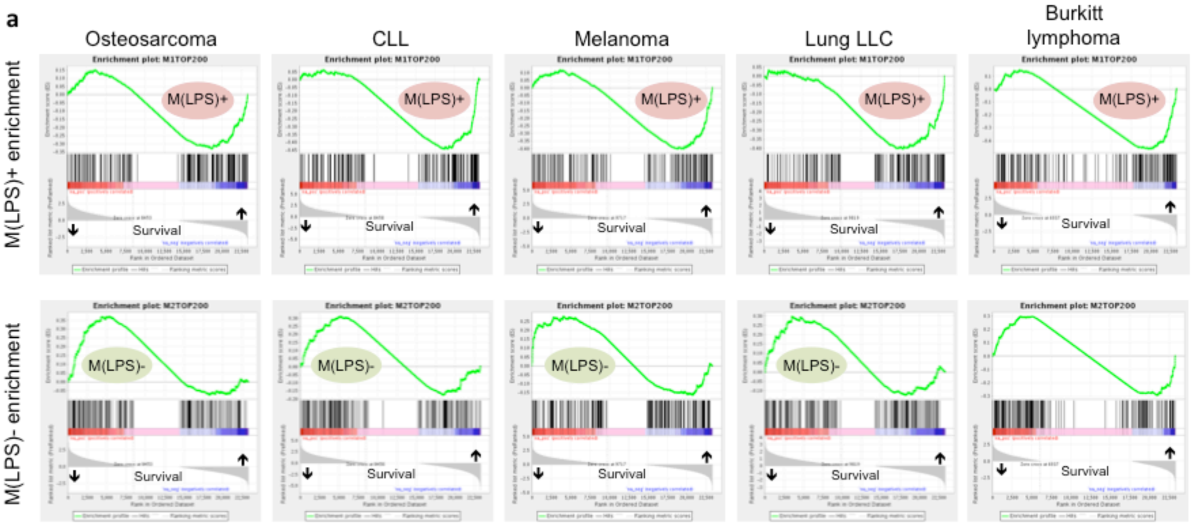

Supplementary Figure 12. Survival analysis in different tumor entities using M(LPS)$^{+/-}$ signatures. Related to Figure 5. a) Gene set enrichment analysis of M1/M2 polarization genes (top 200) in bulk tumor biopsies of the PRECOG dataset (PREdiction of Clinical Outcomes from Genomic Profiles) [2] that ranks genes by patient's survival (left x axis = poor survival, right x axis = favorable survival). Osteosarcoma (M1 p = 0.019, M2 p = 0.007) from GSE39055. Chronic lymphocytic leukemia (M1 p < 0.001, M2 p = 0.189) from GSE22762. Melanoma (M1 p = 0.002, M2 p = 0.290) from SKCM-TCGA. Lung large cell carcinoma (M1 p < 0.001, M2 p = 0.162) from GSE11969. Burkitt lymphoma (M1 p = 0.009, M2 p = 0.986) from GSE4475.

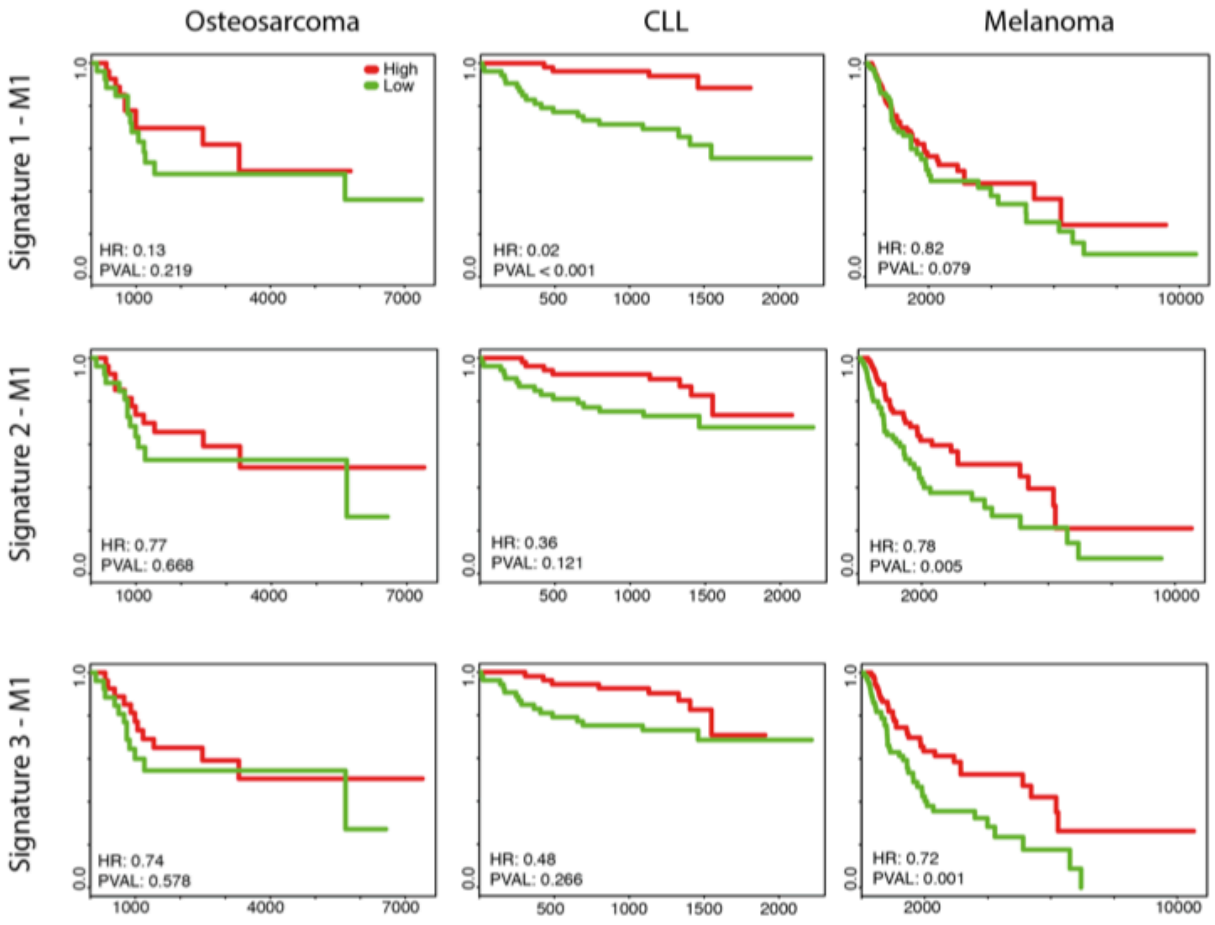

Supplementary Figure 13. Comparison of survival detection between published data sets and M(LPS)$^{+/-}$ signatures. Related to Figure 5. Survival analysis using published gene signatures. Signatures 1-3 are described in Supplementary Figure 9. Hazard ratio and p-value are indicated. Red = high, green = low signature expression. This Figure corresponds to manuscript Figure 5, where the same datasets were analyzed using M(LPS)+ and M(LPS)- signatures. Data from GSE21257 (Osteosarcoma), GSE22762 (Chronic lymphocytic leukemia), and SKCM-TCGA (Melanoma).

Supplementary Table 1

Name	Protocol	Number of genes	Source
Signature 1	C57BL/6 peritoneal macs thioglycollate i.p. 5d<br>- LPS 6h in vitro (M1)<br>- no M2 sig. available	M1: 200 genes	Schroder et al[3]
Signature 2	mouse bone-marrow derived macs<br>- LPS + IFNy 24h (M1)<br>- IL-4 (M2)	M1: 57 genes<br>M2: 33 genes	Jablonski et al[4]
Signature 3	human CD14+ monocyte-derived macs M-CSF polarized<br>- LPS + IFNy or TNFa (M1)<br>- IL-4 or IL-13 (M2)<br>(integrated from three different datasets)	M1: 106 genes<br>M2: 58 genes	Becker M et al[5]

Supplementary References

1. Newman, A. M. *et al.* Robust enumeration of cell subsets from tissue expression profiles. *Nat. Methods* **12,** 453–457 (2015).
2. Gentles, A. J. *et al.* The prognostic landscape of genes and infiltrating immune cells across human cancers. *Nat. Med.* **21,** 938–945 (2015).
3. Schroder, K. *et al.* Conservation and divergence in Toll-like receptor 4-regulated gene expression in primary human versus mouse macrophages. *Proc. Natl. Acad. Sci. U. S. A.* **109,** E944-953 (2012).
4. Jablonski, K. A. *et al.* Novel Markers to Delineate Murine M1 and M2 Macrophages. *PloS One* **10,** e0145342 (2015).
5. Becker, M. *et al.* Integrated Transcriptomics Establish Macrophage Polarization Signatures and have Potential Applications for Clinical Health and Disease. *Sci. Rep.* **5,** (2015).
